# Broadband dual-anisotropic solid metamaterials

**DOI:** 10.1038/s41598-017-13322-2

**Published:** 2017-10-16

**Authors:** Yong Cheng, Xiaoming Zhou, Gengkai Hu

**Affiliations:** Key Laboratory of Dynamics and Control of Flight Vehicle, Ministry of Education and School of Aerospace Engineering, Beijing Institute of Technology, Beijing, 100081 China

## Abstract

We have proposed solid elastic metamaterials with anisotropic stiffness and inertial mass simultaneously, denoted as the dual anisotropy, for the potential use of elastic wave controlling. The dual anisotropy has been designed weakly dispersive in a broad frequency range, wherein broadband anisotropic mass is achieved by employing the sliding-interface concept in fluid-solid composites. Results have been validated through the band-structure, effective-medium, and modal-field analyses. We have further found that the proposed solid metamaterial, when its shear stiffness is diminished until neglected, would reduce to the pentamode-inertial material model. This reduced model is the general form of mediums following transformation acoustic theory, which has been proved vital for acoustic wave controlling. Our studies are expected to pave a new route toward broadband acoustic and elastic wave controlling using dual-anisotropic solid metamaterials.

## Introduction

Undergoing a rapid development in the past decade, metamaterials have shown the great potentials for manipulating acoustic and elastic waves^1–3^. Assisted by the coordinate transformation theory and metamaterial concept, wave-controlling devices designed by artificial gradient microstructures are coming into reality. Anisotropic properties expected from metamaterial building blocks are thought to be pivotal factors for bending wave trajectories in a reflectionless manner. Generally, anisotropic stiffness and anisotropic inertial mass are demanded simultaneously, which is denoted as the dual anisotropy here. In acoustics, the dual anisotropy is a typical manifestation of pentamode-inertial material—the general material model derived from transformation acoustics^4^. For elastic wave control^5,6^, the dual anisotropy is fundamental, not to mention that the Willis couplings^7^ between stress and velocity as well as momentum and strain are additionally needed.

There has been a long history of studies exploiting solid structures with anisotropic elasticity^8,9^. Most of them yet have the isotropic mass, not acting accordingly as wave-controlling mediums requiring dual anisotropy. Distinct from the gravitational mass that is always isotropic, anisotropic mass indicated here refers to the dynamic inertial mass, which attains its tensorial nature from Newton’s second law of motion^10–12^. The inertial mass can be far different from the gravitational one at a finite frequency, being even negative for example, in a strongly dispersive medium with local resonance^13–15^. Following this idea has proposed the conventional resonance concept for making anisotropic mass. It means to introduce different resonant frequencies at various directions of composite’s building blocks^10,16^. Practical structure models have been extensively studied later, including the modified spherical particulate composites with elliptical cores or elliptical coatings^17–19^, and so on^20–22^. However, anisotropic mass acquired near the resonance is accompanied with the strong dispersion, resulting in inevitably a narrow band of operating frequency. The challenge for designing the dual-anisotropic solid metamaterials is to broaden the anisotropic-mass bandwidth, as is currently unavailable through the concept of resonance.

In contrast to all-solid metamaterials described above, fluid-solid composites are employing a non-resonant mechanism for making the anisotropic density that is dispersionless inherently^23–27^. It is caused substantially by the discontinuity or sliding effect of particle tangential motion at the fluid-solid interface. This distinct mechanism has resulted in unusual density laws unlikely observed in all-solid realms. For example, alternating (A/B/A…) layered fluid-solid composites possess the anisotropic density that reads $1/{\rho }_{{\rm{t}}}={c}_{{\rm{A}}}/{\rho }_{{\rm{A}}}+{c}_{{\rm{B}}}/{\rho }_{{\rm{B}}}$ along the direction tangential to the layer surface, and ${\rho }_{{\rm{n}}}={c}_{{\rm{A}}}{\rho }_{{\rm{A}}}+{c}_{{\rm{B}}}{\rho }_{{\rm{B}}}$ in the surface normal, where ${c}_{{\rm{A}},{\rm{B}}}$ indicates the volume fraction^28^. As another example, effective density of a fluid host embedded with solid particles is governed by the Berryman’s formula^29,30^, rather than the intuitive volume-averaged density. Anisotropic inertial mass created by the sliding-interface concept is dispersionless in a broad band ranging from quasi-statics. These fluid-solid composites are also called metafluids incapable of the shear resistance, and preferable for broadband acoustic control in fluid or gas. They have been intensively exploited in order to design acoustic cloaks^31,32^, sub-diffraction limited imaging devices^33,34^, and metasurfaces for the unprecedented wavefront modulation^35^, etc. The non-resonant sliding-interface concept appears as a feasible route towards broadband inertial anisotropy in metafluids. However, it remains rarely explored how the sliding-interface concept is implemented in all-solid structured materials that permit only fully bonded interfaces. We would make an attempt in this work.

## Results

### Metamaterial design

**Figure 1:**
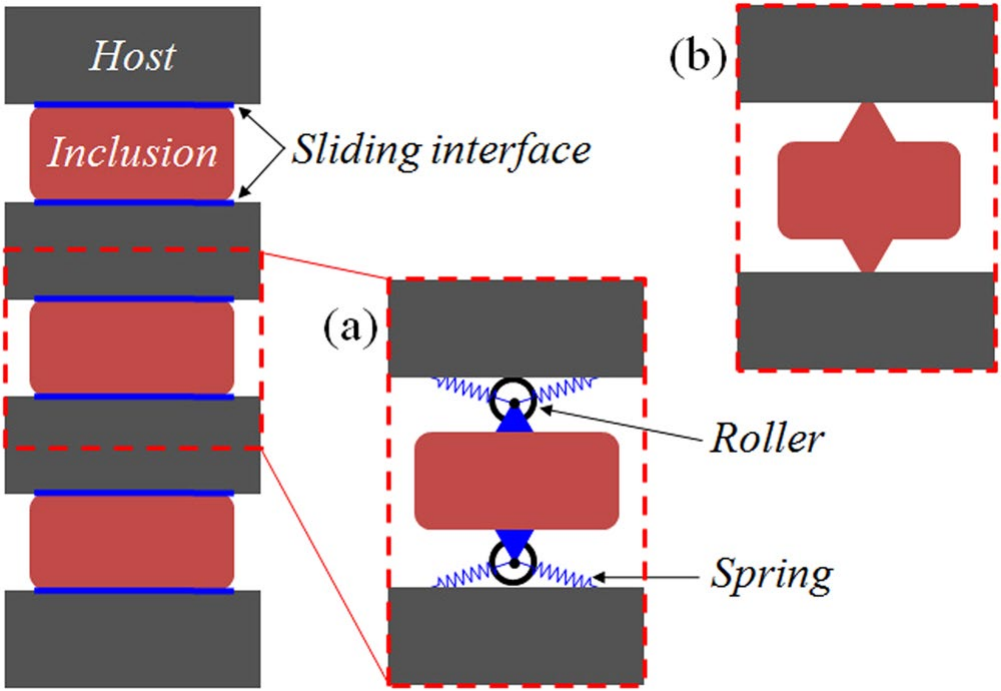
Conceptual models of solids with broadband anisotropic mass. (**a**) A layered solid-solid structure with the sliding interfaces and one of phases constitutes the host, wherein a moderate interface stiffness, denoted by the spring, needs to be added in order for the mechanical stability. (**b**) A realistic structural material that is designed according to the model in (**a**) where the spring-reinforced sliding interface is practically realized by the slender geometric connection between layered solids.

Consider a layered solid-solid structure, wherein the sliding boundary is imposed for all interfaces and one of phases constitutes the host, as shown schematically in Fig. 1. It is apparent that the overall inertial density is anisotropic. The vertical mass-component measures the total weight owing to the uniform motion in composite, while the horizontal one describes merely the host’s weight due to the slipping effect. Anisotropic mass attained in this model has inherited the motion-discontinuity feature of metafluids and therefore been dispersionless. However, structured materials containing the sliding interface, which can be represented by rollers running at the frictionless boundary as shown in Fig. 1(a), is mechanically unstable, since the internal structures would slip away. To ensure the stability, a moderate interface stiffness denoted by the springs needs to be introduced. This would inevitably induce a low-frequency resonance. Nevertheless, the nearly dispersionless anisotropic density can still be obtained in a broad frequency range beyond the resonance, as will be verified in this work. From a practical point of view, the slender joints at interfaces between layers act pertinently as rollers with a minor spring reinforcement, as schematically shown in Fig. 1(b). In the following, we base this structural model to propose a practical version of broadband dual-anisotropic metasolids.

**Figure 2:**
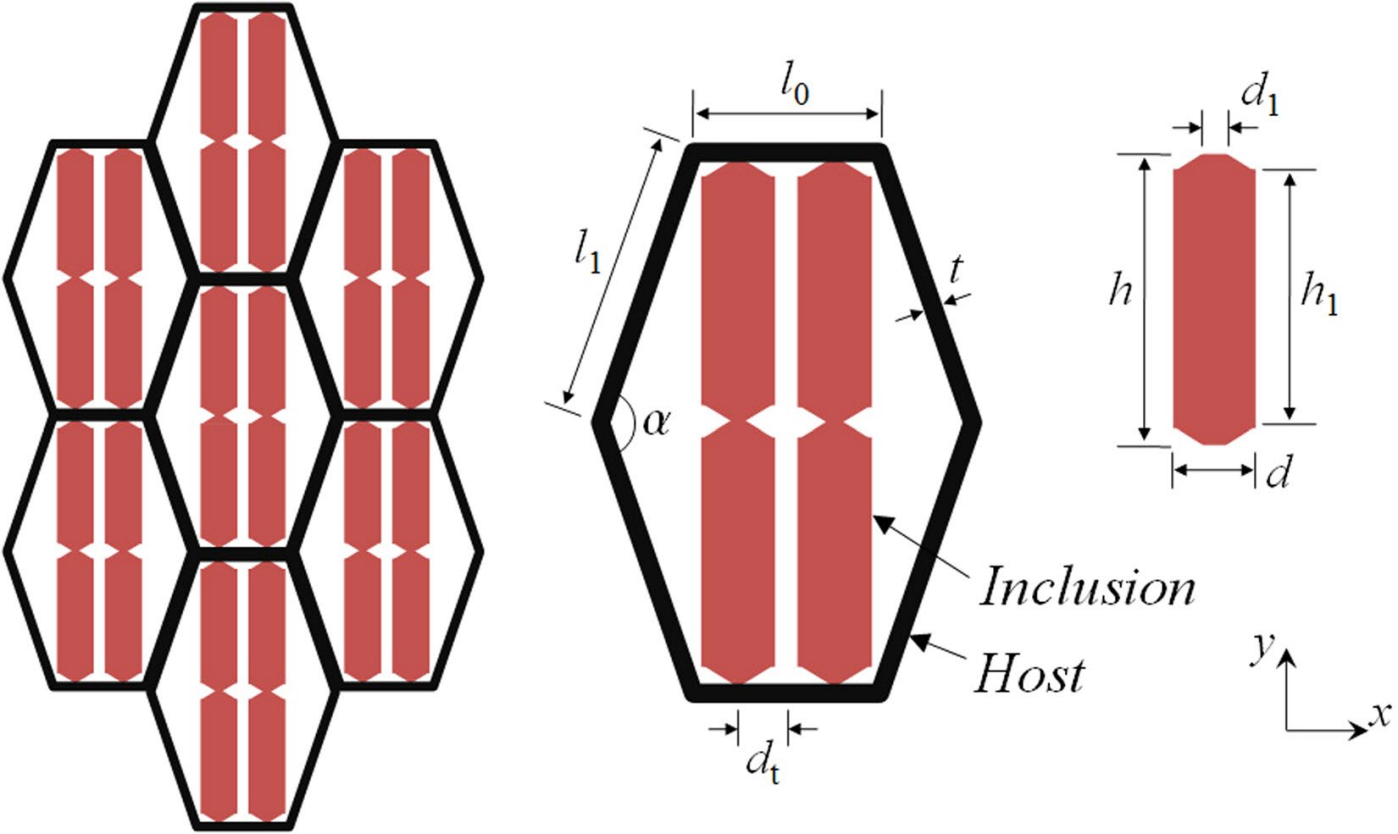
Realistic model of the dual-anisotropic structured material. The model consists of a periodic hexagonal lattice as the host, governing the overall anisotropic stiffness, and two bars as the inclusion, achieving the anisotropic inertial density. Slender cross-sections are made at both the middle and two ends of bars in order to simulate the sliding boundary in fluid-solid interfaces, meanwhile keep the structure mechanically stable. Structural parameters are *l*_0_ = 2*l*_1_/3 = 8*t* = 10 mm and α = 140° for the lattice host, and *d* = 10*d*_1_ = 4 mm, *d*_t_ = 2.15 mm, and *h*_1_ = 0.8 *h* for the inclusion.

The proposed dual-anisotropic 2D metasolid consists of a periodic hexagonal lattice as the host, connected internally to two vertically oriented bars, which have been sharpened at both the middle and two ends, as shown in Fig. 2. Broadband anisotropic density is to be realized due to the sharpened bar inclusions, wherein the slender cross-sections are made for pursuing a very small connecting stiffness in order to simulate the sliding-boundary effect. In addition, it is critical to choose the proper inclusion material having preferably the low stiffness for the minimum coupling to the lattice strut, the large density for the high anisotropic-density ratio, and the low loss that is essential in practical applications. The inclusion material suggested here is the low-loss fiber-glass filled fluorinated ethylene propylene^36^ with Young’s modulus *E*_Y_ = 1.7 GPa, Poisson’s ratio *v* = 0.4, and density ρ = 2200 kg/m^3^. The lattice host is made by aluminum (*E*_Y_ = 71 GPa, *v* = 0.33, and ρ = 2700 kg/m^3^) and functions primarily for the overall anisotropic elasticity^9^, which can be modulated by four independent geometric parameters, which are the lengths *l*_0_ for the beam directed horizontally and *l*_1_ for the others, the thickness *t* for all, and the included angle *α* between two adjacent declining beams. Notice that the lattice geometry employed here was considered previously for the host structure of pentamode materials^37^, which has been demonstrated possessing anisotropic mechanical properties and fluid-like elasticity in a broad frequency band. Consequently, the present model holds the potentials of being extended to the generalized pentamode-inertial materials, which would possess not only the anisotropic compressibility as usual, but also the broadband anisotropic mass newly developed here, as will be discussed in the final part of the study. Below, band structure calculations and effective medium analyses will be performed in order for fully decoding wave signatures of the proposed metasolid.

### Band-structure and effective-medium analyses

**Figure 3:**
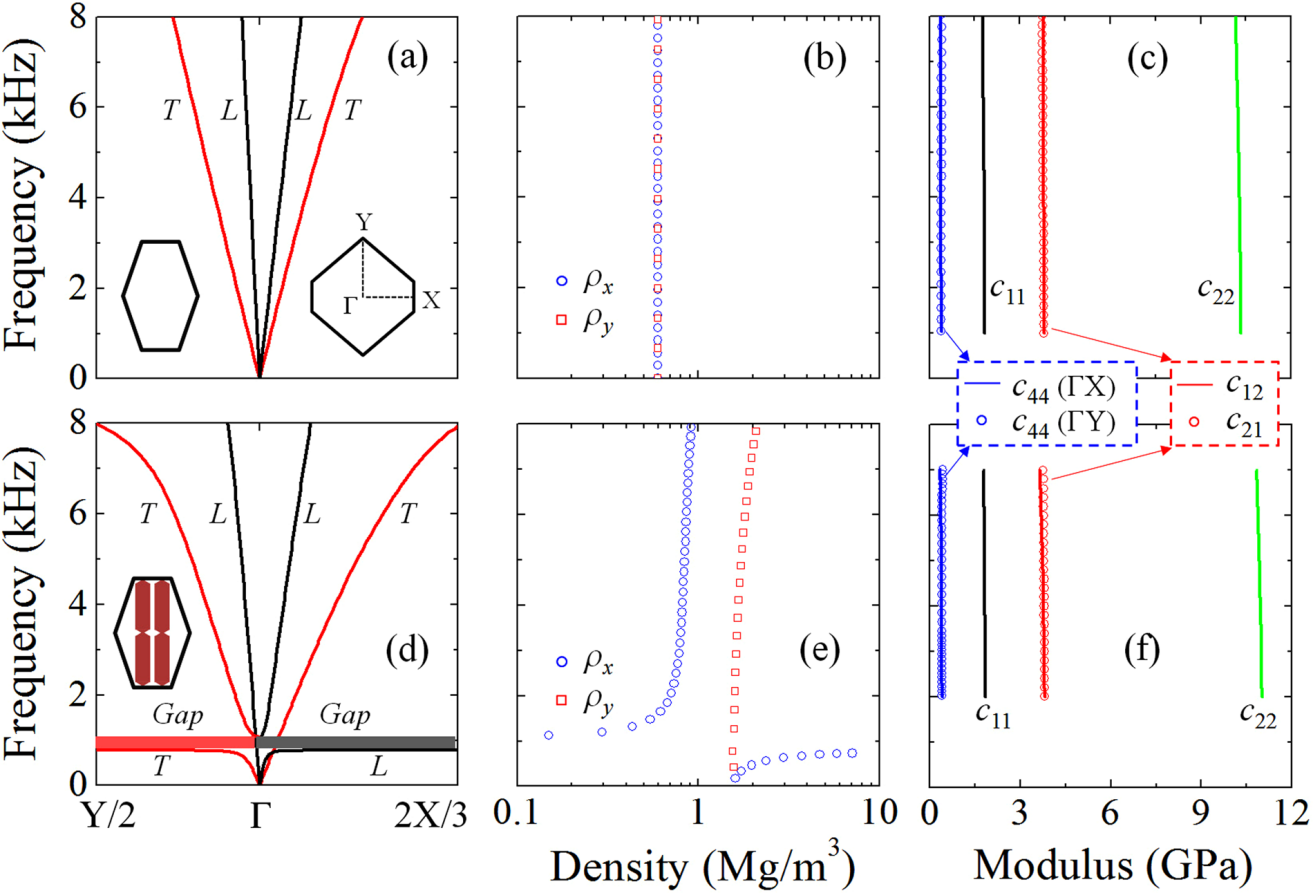
Wave characteristics of the dual-anisotropic material. Band structures of the pure lattice unit (**a**) and the metasolid cell (**b**) in two perpendicular ΓX and ΓY directions, whose definition in the first Brillouin zone is shown in the inset. (**b**) Effective inertial densities and (**c**) stiffness parameters retrieved for the lattice host. (**e**), (**f**) Effective-medium parameters of the metasolid, showing that weakly dispersive dual anisotropy can be achieved in a broad frequency range ~2.0–6.0 kHz.

We examine firstly dispersion characteristics of the pure lattice without containing the inclusion. Figure 3(a) shows its band structure in two perpendicular ΓX and ΓY directions, whose definition in the first Brillouin zone is plotted as the inset. In either ΓX or ΓY directions, two dispersionless bands can be observed and adhered to the longitudinal (L) mode with a high phase velocity and the transverse (T) mode with a lower one, as recognized by the associated eigenfunctions. The L modes are asymmetric in *x* and *y* directions, implying the overall anisotropic property of the lattice strut. These dispersionless and asymmetric branches are always available in a finite band far below the zone boundary. For the metasolid cell containing the inclusion, the band structure in Fig. 3(d) shows that, in the ΓX direction, the L branch is broken by a gap near 1 kHz, accompanying there a strongly distorted dispersion, while the T branch remains uninterrupted. The similar phenomenon appears in the ΓY direction, yet has been reversed, showing that the T branch is now interrupted at the same gap frequency, whereas the L branch remains continuous. It can be seen that the linear dispersion of the host, has been distorted by the gap when the inclusion is added, yet recovered immediately beyond the gap and sustained in a broad frequency range. Based on an effective medium model developed in the following for the dual-anisotropic solid, we will demonstrate that asymmetric L branches for the pure lattice is due to the anisotropic stiffness only, while the anisotropic density has been additionally realized in that recovered linear dispersion regime when the inclusion is added.

From the unit cell geometry, it is straightforward to see that there exist two mutually perpendicular symmetry planes in alignment with the ΓX and ΓY directions. Thereby, the structured metasolid is best represented by the continuum with simultaneously the orthotropic stiffness and density. The constitutive equation governing the orthotropic elasticity is given by

1 $$[\begin{array}{c}{\sigma }_{xx}\\ {\sigma }_{yy}\\ {\sigma }_{xy}\end{array}]=[\begin{array}{ccc}{c}_{11} & {c}_{12} & 0\\ {c}_{21} & {c}_{22} & 0\\ 0 & 0 & {c}_{44}\end{array}]\,[\begin{array}{c}{\varepsilon }_{xx}\\ {\varepsilon }_{yy}\\ 2{\varepsilon }_{xy}\end{array}].$$

In order to retrieve all elastic parameters, the stress and stain fields, ${\sigma }_{\alpha \beta }$ and ${\varepsilon }_{\alpha \beta }$ (*α*, *β* = *x* or *y*), are obtained as the local field integration over a single cell^38^. To be more specific, the average normal stress and normal strain are defined from the L modes and then used to compute uniquely four elastic constants *c*_11_, *c*_12_, *c*_21_, and *c*_22_ according to ${[{\bar{\sigma }}_{xx},{\bar{\sigma }}_{yy}]}_{{\rm{LX}}}=[({c}_{11},{c}_{12}),({c}_{21},{c}_{22})]{[{\bar{\varepsilon }}_{xx},{\bar{\varepsilon }}_{yy}]}_{{\rm{LX}}}$ and ${[{\bar{\sigma }}_{xx},{\bar{\sigma }}_{yy}]}_{{\rm{LY}}}=[({c}_{11},{c}_{12}),({c}_{21},{c}_{22})]{[{\bar{\varepsilon }}_{xx},{\bar{\varepsilon }}_{yy}]}_{{\rm{LY}}}$. Average shear stress and shear strain are defined from the T modes and the resultant shear modulus *c*_44_ is computed by either ${({\bar{\sigma }}_{xy})}_{{\rm{TX}}}={c}_{44}{(2{\bar{\varepsilon }}_{xy})}_{{\rm{TX}}}$, or ${({\bar{\sigma }}_{yx})}_{{\rm{TY}}}={c}_{44}{(2{\bar{\varepsilon }}_{yx})}_{{\rm{TY}}}$. On the other hand, the equation of inertial motion considering the anisotropic density reads

2 $$[\begin{array}{c}{F}_{x}\\ {F}_{y}\end{array}]=-{\omega }^{2}[\begin{array}{cc}{\rho }_{x} & 0\\ 0 & {\rho }_{y}\end{array}]\,[\begin{array}{c}{u}_{x}\\ {u}_{y}\end{array}],$$

where the net force ${F}_{\alpha }$ and displacement ${u}_{\alpha }$ are again the averaging fields over the cell^38^ by using the eigenfunction of L modes. Ultimately, density components are calculated from ${\rho }_{x}=(-1/{\omega }^{2}){({F}_{x})}_{{\rm{LX}}}/{({\bar{u}}_{x})}_{{\rm{LX}}}$ and ${\rho }_{y}=(-1/{\omega }^{2}){({F}_{y})}_{{\rm{LY}}}/{({\bar{u}}_{y})}_{{\rm{LY}}}$.

We have first computed effective-medium parameters of the pure lattice, as shown in Fig. 3(b) and (c). It is found that the density is an isotropic one ~598 kg/m^3^, which is exactly the static gravitational one as expected. Anisotropic effective stiffness is observed, namely ${c}_{11}\ne {c}_{22}$, which accounts for the unsymmetrical L branches in ΓX and ΓY directions. Note that the retrieved off-diagonal components *c*_12_ and *c*_21_ are equal exactly, and in addition, *c*_44_ is unique regardless of being determined from LX or LY modes. These are the evidences verifying the Cauchy continuum nature of structured materials. Retrieved effective density and stiffness of the metasolid are shown respectively in Fig. 3(e) and (f). In comparison to the pure lattice, the reinforcement effect of elasticity is minor due to the added ‘soft’ inclusion. The distinction is revealed by the variation of density. We observe that ${\rho }_{y}$ is nearly the composite average density ~1563 kg/m^3^, whereas ${\rho }_{x}$ agrees with ${\rho }_{y}$ at statics, but converging quickly to a smaller constant ~820 kg/m^3^ after passing through a strong resonance-like fluctuation zone, which is relevant to the bandgap region. Results clearly demonstrate that the gap-related modulation that arises from the added inclusion plays a vital role in the transition from isotropic density of the host lattice to anisotropic one of the metasolid. The anisotropic density achieved here is nearly dispersionless in a broad frequency range ~2.0–6.0 kHz, which corresponds well to the recovered linear dispersion regime in band diagrams.

### Analysis of modal fields

**Figure 4:**
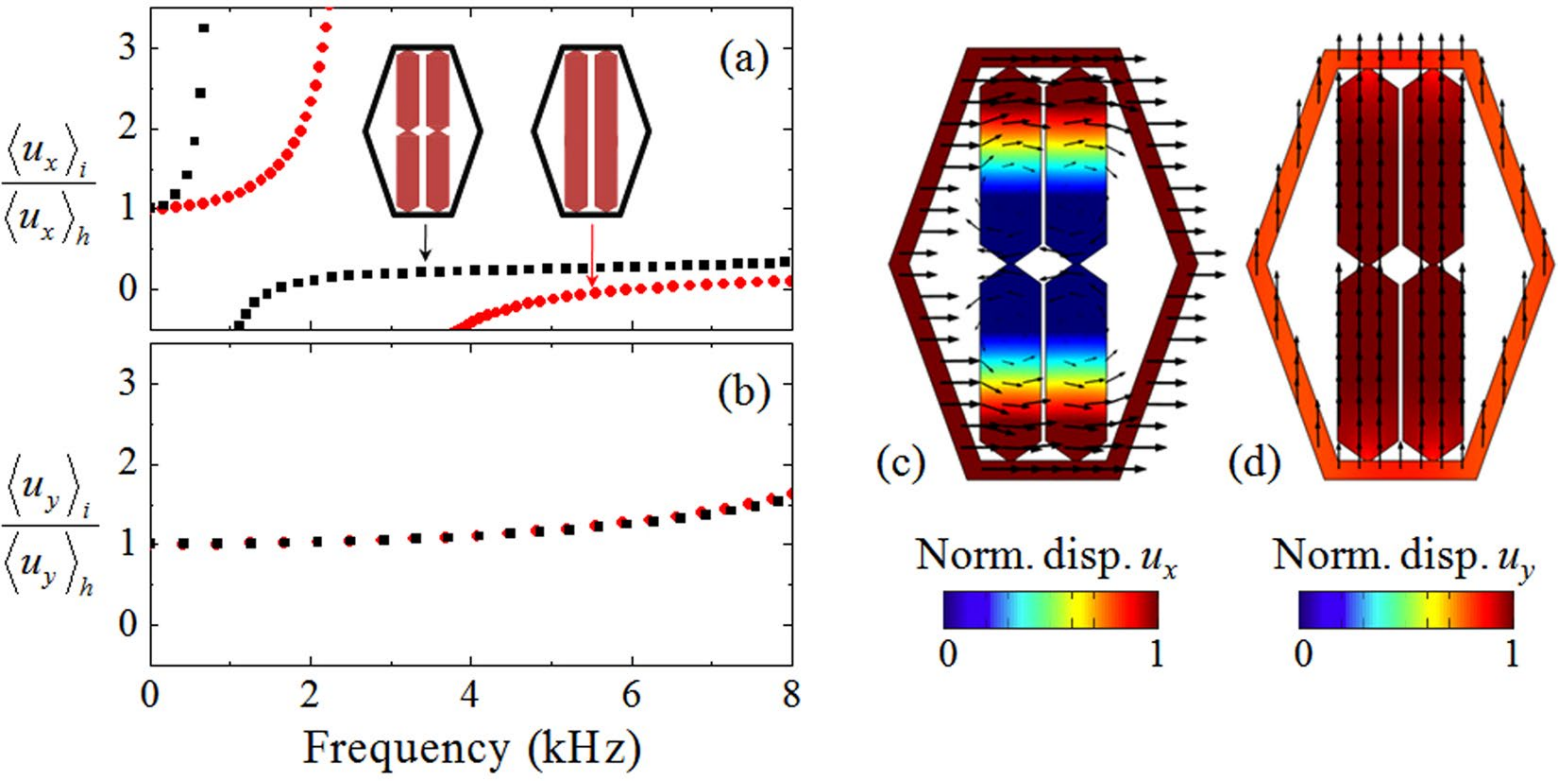
Displacement field distributions of some specific eigenstates. Average displacements of the inclusion with respect to the lattice host, (**a**) ${\langle {u}_{x}\rangle }_{i}/{\langle {u}_{x}\rangle }_{h}$, and (**b**) ${\langle {u}_{y}\rangle }_{i}/{\langle {u}_{y}\rangle }_{h}$, calculated respectively from LX and LY band diagrams. Mode shapes of displacement fields (**c**) *u*_*x*_ and (**d**) *u*_*y*_ at a certain frequency 3 kHz that is inside the linear dispersion regime. (**c**) The ${u}_{x}$ field verifies that the central region of the inclusion remains almost motionless, mimicking the sliding-boundary effect in fluid-solid composites. The slender cross-section carved at the middle of the bar helps to further diminish the resonant frequency as verified by a comparison to the straight-bar case. (**d**) Uniform displacement distribution ${u}_{y}$ observed in the LY mode explains why ${\rho }_{y}$ reaches the composite average density.

Deeper physical insights into broadband anisotropic density can be gained by analyzing average displacements of the inclusion with respect to the lattice host, ${\langle {u}_{x}\rangle }_{i}/{\langle {u}_{x}\rangle }_{h}$, and ${\langle {u}_{y}\rangle }_{i}/{\langle {u}_{y}\rangle }_{h}$, which are retrieved respectively from LX and LY branches, as shown in Fig. 4(a) and (b). For more details, the modal displacement fields of LX and LY branches at a certain frequency 3 kHz that is inside the linear dispersion regime are shown in Fig. 4(c) and (d). Here, the LX branch indicates the mode of the cell structure oscillated horizontally. The ${u}_{x}$ field in Fig. 4(c) reveals that, due to the slender connection made at both ends of bars, the central region of the inclusion remains almost motionless, mimicking the sliding-boundary effect in fluid-solid composites. The slender connection between the host and inclusion acts as a soft spring, which together with the inclusion’s mass effect comprises a mechanical resonator. This explains the fact that the spectrum profile of ${\rho }_{x}$ in Fig. 3(e) follows approximately the Lorentz dispersion model $\bar{{\rm{w}}}={{\rm{w}}}_{m}+{{\rm{w}}}_{i}{\omega }_{0}^{2}/({\omega }_{0}^{2}-{\omega }^{2})$, wherein ${{\rm{w}}}_{i}$ and ${{\rm{w}}}_{m}$ resemble respectively the weight of the inclusion and host, and the resonant frequency ${\omega }_{0}$ is associated to the connecting stiffness between them. We can then understand the broadband anisotropic densities through the Lorentz dispersion characteristics. It means that perfect sliding boundary corresponds to the case of zero connecting stiffness, namely ${\omega }_{0}=0$, hence a dispersionless mass $\bar{{\rm{w}}}={{\rm{w}}}_{m}$ is acquired, which is irrelevant to the weight of inclusion. In the nonideal case with a small boundary stiffness ${\omega }_{0}\to 0$, which is practically needed in order to ensure the motion stability of inclusions, the nearly dispersionless density $\bar{{\rm{w}}}\approx {{\rm{w}}}_{m}$ can still be achieved at frequencies far beyond ${\omega }_{0}\approx 0$, as is the case of our model. It is worth to stress that, the slender cross-section carved at the middle of the bar is essential to decrease further the resonant frequency ${\omega }_{0}$, as evidenced by the comparison result in Fig. 4(a).

Figure 4(d) shows the displacement field ${u}_{y}$ of the LY mode, which is linked to the structure vibration along the vertical direction. Uniform displacement distribution is observed and accounts for why ${\rho }_{y}$ measures the average density of the whole composite. The result states that the internal bars, though sharpened at ends and the middle, yield still a strong bonding along the bar axial direction. According to the Lorentz model, the strong bonding means ${\omega }_{0}\to +\infty$, and then causes a dispersionless density $\bar{{\rm{w}}}\approx {{\rm{w}}}_{m}+{{\rm{w}}}_{i}$ at frequencies far below ${\omega }_{0}\approx +\infty$. The Lorentz model has shown that the limiting anisotropic ratio of densities in metasolids reads $({{\rm{w}}}_{m}+{{\rm{w}}}_{i})/{{\rm{w}}}_{m}$. The strategy to enlarge the anisotropic ratio is to choose heavy inclusion and lightweight host structure. The anisotropic ratio approaches around 1.9 in our example, but can be definitively enhanced by weighting the inclusion through, for example, adding heavy attachments.

### Pentamode-inertial material model

**Figure 5:**
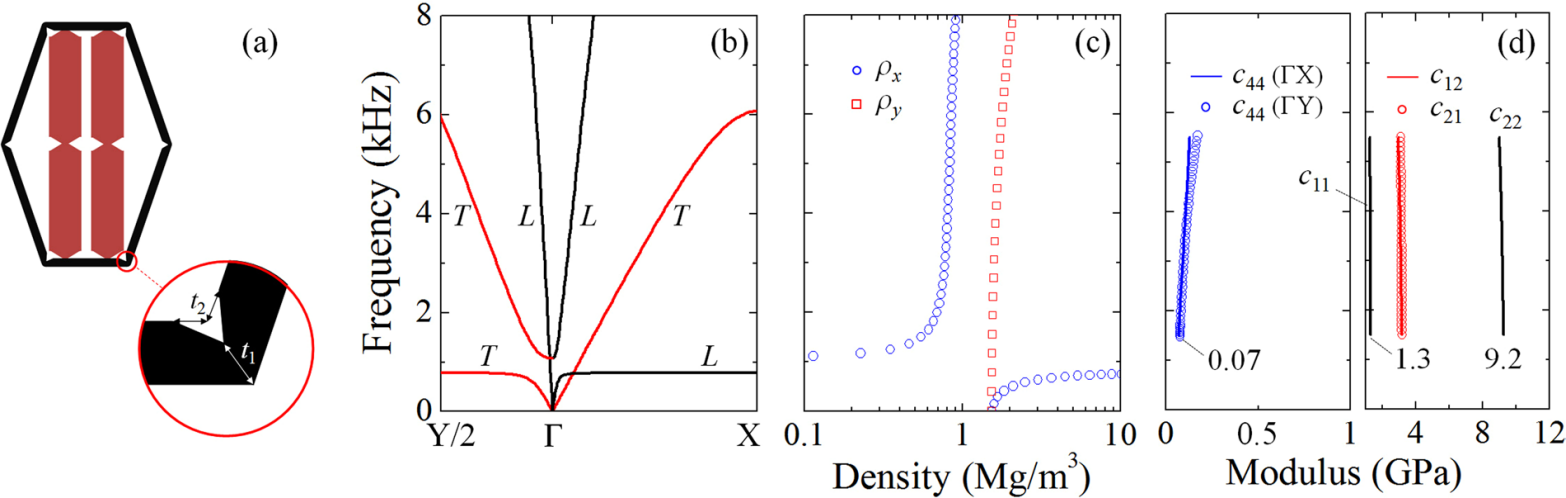
Realistic model and wave characteristics of pentamode-inertial materials. (**a**) The reduced model of the metasolid wherein all lattice beams are sharpened at ends with parameters *t*_1_ = *t*_2_ = *t*/2; (**b**) band structures, (**c**) effective inertial densities, and (**d**) overall stiffness parameters computed for the modified model. This reduced metasolid, whose shear stiffness *c*_44_ has been diminished remarkably, behaves like the pentamode-inertial material, which is the general material model following transformation acoustic theory.

Study further a reduced model of the proposed metasolid, which is modified with a sufficiently small shear stiffness *c*_44_. To achieve that, we simply sharpens the ends of all six strut beams in a lattice unit, and keep the inclusion’s geometry unaltered, as shown in Fig. 5(a). Band diagrams plotted in Fig. 5(b) show that the linear dispersion is still achieved in a broad frequency range beyond the resonance gap. The distinction is obviously revealed, wherein the slope of the linear T modes is lowered greatly, while the slope is dropped down slightly for L modes. Results are in good accordance with the effective-medium predictions, in which the shear stiffness *c*_44_ has been decreased remarkably to 0.07 GPa, while *c*_11_ and *c*_22_ are lowered by a small amount to 1.3 and 9.2 GPa respectively, as evidenced in Fig. 5(c). In addition, there is almost no change for broadband anisotropic densities upon the geometric variation of the lattice beams. The model geometry considered here hasn’t been optimized; one can diminish further the connection thickness *t*_1_, until *c*_44_ can be neglected from a practical point of view. Consequently, the metasolid having a negligible shear resistance has reduced to the pentamode-inertial (PI) material—the solid material with the fluid-like elasticity and anisotropic density. The PI material has been recognized as a general model following transformation acoustic theory^4^. It appears to be the first time that the structured model of PI materials is practically designed in our study, which may open a new avenue for acoustic wave controlling.

## Discussions

The proposed dual-anisotropic metamaterial is of the host-inclusion type, consisting of the stiff hexagonal lattice in which the soft two-bar inclusions are embedded. The overall anisotropic stiffness is fully governed by the lattice strut, and is nearly irrelevant to longitudinal, bending, or buckling deformations of bars since the inclusion material is very soft. The sharpened bar inclusion is designed by mimicking the sliding-boundary effect in fluid-solid composites in order to pursue the broadband anisotropic density. Effective medium methods of metamaterials have been developed, making the micro-macroscopic relationships clearly identified. Note that the proposed solid metamaterials with dual anisotropy, if the Willis couplings are further incorporated, would fulfill the requirement of transformation elastic theory^5,6^. However, under some special forms of coordinate transformations, for example in the case of linear transformation, the Navier elastodynamic equation retains its original form. It is the case where elastic wave controlling can be made possible by using our dual-anisotropic materials without requesting Willis coupling effect.

In our model, the dynamic coupling between the host and soft inclusion is very weak, allowing us to modulate the overall stiffness without influencing anisotropic densities. We have then proposed a significant reduced model by diminishing the shear stiffness of the metasolid, which is the so-called pentamode material with anisotropic inertial. Pure pentamode materials^39–42^ with the anisotropic stiffness only, suggested firstly by Milton and Cherkaev^37^, have only one non-zero elastic eigenvalue that is of the hydrostatic stress state, exhibiting therefore the fluid-like elasticity. They belong to a special scenario of transformed acoustic equations^43,44^. The most general material form enabled by transformation acoustics under an arbitrary coordinate transformation refers to the PI material as realized here. All of our model studies have been validated through band-structure, effective-medium, and modal-field analyses. Results are expected to make an important step toward acoustic and elastic wave controlling with important applications to cloaking, seismic protection, and shock mitigation.

### Data Availability

The datasets generated during and/or analysed during the current study are available from the corresponding author on reasonable request.
